# Paclitaxel Through the Ages of Anticancer Therapy: Exploring Its Role in Chemoresistance and Radiation Therapy

**DOI:** 10.3390/cancers7040897

**Published:** 2015-12-03

**Authors:** Anna Maria Barbuti, Zhe-Sheng Chen

**Affiliations:** Department of Pharmaceutical Sciences, College of Pharmacy and Health Sciences, St. John’s University, Queens, NY 11439, USA; anna.barbuti13@stjohns.edu

**Keywords:** paclitaxel, cancer, chemotherapy, multidrug resistance, chemoradiotherapy

## Abstract

Paclitaxel (Taxol^®^) is a member of the taxane class of anticancer drugs and one of the most common chemotherapeutic agents used against many forms of cancer. Paclitaxel is a microtubule-stabilizer that selectively arrests cells in the G2/M phase of the cell cycle, and found to induce cytotoxicity in a time and concentration-dependent manner. Paclitaxel has been embedded in novel drug formulations, including albumin and polymeric micelle nanoparticles, and applied to many anticancer treatment regimens due to its mechanism of action and radiation sensitizing effects. Though paclitaxel is a major anticancer drug which has been used for many years in clinical treatments, its therapeutic efficacy can be limited by common encumbrances faced by anticancer drugs. These encumbrances include toxicities, *de novo* refraction, and acquired multidrug resistance (MDR). This article will give a current and comprehensive review of paclitaxel, beginning with its unique history and pharmacology, explore its mechanisms of drug resistance and influence in combination with radiation therapy, while highlighting current treatment regimens, formulations, and new discoveries.

## 1. Introduction

**Figure 1:**
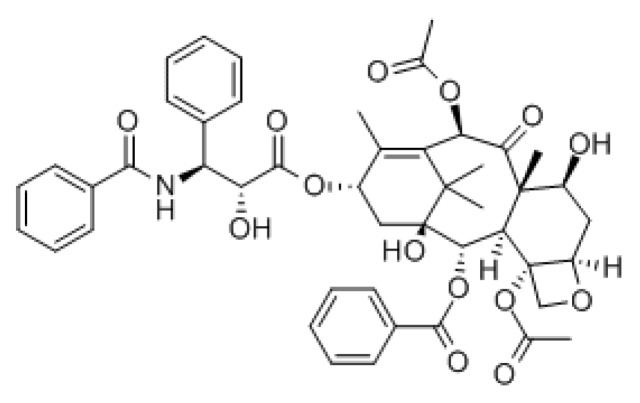
Chemical structure of paclitaxel.

Paclitaxel (Figure 1) was discovered as part of a National Cancer Institute screening program, initiated in 1960 under Dr. Jonathan L. Hartwell, in search for plant extracts with antineoplastic activity [1]. Found in the bark extract of the Pacific Yew Tree, *Taxus brevifolia*, the isolation and identification of paclitaxel (which they named Taxol^®^) was accomplished by Drs. Wall and Wani, and published in 1971 [2]. Still, it took some years before paclitaxel’s mechanism of action as an antitumor drug was of interest to the cancer pharmacology world, or even to the pharmaceutical companies. The laboratory of Dr. Horwitz investigated and verified that paclitaxel not only had potent cytotoxic properties able to inhibit the growth of human cervical cancer cells (HeLa) at nanomolar concentrations, but also that paclitaxel arrested cells in the mitotic (M) phase of the cell cycle, without disruption of the synthesis (S) phase [3,4]. Further biochemical assays and experimentations were studied by scientists to uncover the profound and unique properties of paclitaxel [5]. Decades later, investigations of paclitaxel as an anticancer agent are still being conducted. 

## 2. The Pharmacology of Paclitaxel

Microtubules are a dynamic network of tubulin heterodimers, of α and β subunits, involved in many important cellular processes, especially in making up the mitotic spindle fibers necessary for M phase cell division [6]. The earlier microtubule targeting drugs, such as colchicine and the vinca alkaloids, induce the disassembly of microtubules. Conversely, the taxanes bind to tubulin and inhibit the disassembly of microtubules. The antimicrotubule agents, whether functioning to depolymerize or stabilize tubulin, disrupt the microtubule dynamics, induce mitotic arrest and prohibit cell division to ultimately cause apoptosis. 

Paclitaxel is a member of the taxane family of anticancer drugs, along with docetaxel. Though the main induction of apoptosis is by targeting tubulin, paclitaxel has also been found to target the mitochondria and inhibit the function of the apoptosis inhibitor protein B-cell Leukemia 2 (Bcl-2) [7]. Paclitaxel is a strongly hydrophobic drug, which requires suitable delivery vehicles to effectively distribute into tumor tissues. For efficient distribution of this hydrophobic anticancer drug, paclitaxel is currently formulated and administered to patients via polyethoxylated castor oil (Cremophor EL, CrEL) or albumin-bound (nab-paclitaxel, Abraxane^®^). Intravenous (IV) administration of CrEL-paclitaxel, usually given once every 3 weeks, has been the classic route for many years, but reported as causing hypersensitivity reactions and neurotoxicity [8]. To decrease the toxicity and enhance delivery and distribution, new paclitaxel formulations involving the use of nanoparticles, emulsions, liposomes, and micelles have been explored [9]. Nab-paclitaxel, a newer albumin-bound paclitaxel nanoparticle form, has been found to lessen hypersensitivity reactions because it does not contain CrEL. In fact, Li *et al.* reported the differences in paclitaxel distribution between CrEL- and nab-paclitaxel. The distribution of nab-paclitaxel to peripheral tissue was faster (4-fold) and more extensive (10-fold) when compared to CrEL-paclitaxel micelles [10]. The study also noted that tissue distribution of free and protein bound paclitaxel, when released from the respective carrier complexes, was limited and slow, attesting to the drug’s hydrophobic nature. 

The taxanes are metabolized in the liver by the cytochrome P450 enzymes and are eliminated by biliary excretion [11]. The known metabolites of paclitaxel are generally inactive when biotransformed via hydroxylation reactions. These metabolites include 6-α-hydroxypaclitaxel (by CYP2C8), 3′-*p*-hydroxyphenylpaclitaxel (by CYP3A4) and 6-α-p-3-dihydroxypaclitaxel (by further metabolism of the prior metabolites via CYP3A4 and CYP2C8, respectively) [12]. The toxicities associated with paclitaxel are primarily neutropenia, as well as peripheral neuropathy and some cardiotoxicity [11]. Still, due to the fact paclitaxel is excreted through the bile, it is often the preferred anticancer therapy for patients with impaired creatinine clearance or renal disease. The ATP Binding Cassette (ABC) transporter B1 (ABCB1/MDR1/P-gp) also has been found to play a role in the metabolism of paclitaxel [8,13]. However, the role of ABCB1 in paclitaxel-treated cells is known to be more closely connected with drug resistance which will be discussed further in this review.

Paclitaxel has been approved by the FDA to be used alone, or in combination with other anticancer treatments, to treat AIDS-related Kaposi sarcoma, breast cancer, non-small cell lung cancer (NSCLC), and ovarian cancer. It is also being studied and can be used to treat many other cancers including head and neck, esophagus, bladder, endometrial and cervical cancer. The FDA has also more recently approved Abraxane^®^ to treat metastatic pancreatic cancer, non-small cell lung cancer, and breast cancer. The administration warning included with these paclitaxel formulations is to monitor patient neutrophil counts to manage any bone marrow suppression [14]. Chemotherapy-induced neutropenia is a common adverse effect of many anticancer drugs.

## 3. Paclitaxel in Drug Resistance 

**Figure 2:**
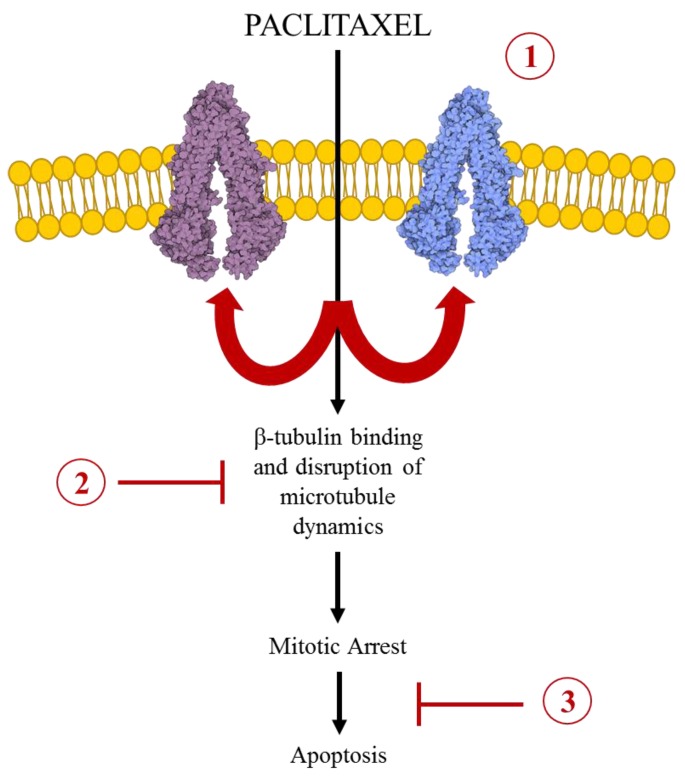
The Major Mechanisms of Paclitaxel Resistance. The cellular mechanism of action by which paclitaxel serves as an anticancer drug, as illustrated following the black arrows. Paclitaxel enters the cell and binds to b-tubulin on the inner surface of microtubules. This stabilizes the microtubule network, arrests the cell cycle at the G2/M phase, and therefore leads to apoptosis. Cancer cells have been found to evade the microtubule stabilizing action of paclitaxel through three main mechanisms (illustrated in red): (1) over-expression of transmembrane efflux transporters, specifically ABCB1 and ABCC10; (2) tubulin mutations (both α and β) or alterations in the stability of the microtubule network; and (3) reduced function of significant apoptotic proteins, such as Bcl-2 and p53.

One of the major caveats to anticancer treatments is the development of drug resistance. Cellular and molecular mechanisms which contribute to chemoresistance include: alterations in membrane lipids, compartmentalization (in endocytic vesicles), induction of emergency response elements, altered cell cycle checkpoint proteins, an increase or alteration in drug targets, metabolism (bio-inactivation), inhibited apoptotic responses (Bcl-2), increased DNA damage repair, decreased uptake (through downregulation of a receptor), and increased efflux (by overexpressed ABC transporters) [15]. Specifically, paclitaxel has been found to induce multidrug resistance (MDR) through manipulation of cellular mechanisms including: overexpression of the ATP-binding cassette (ABC) transporters, alterations in binding regions of β-tubulin and tubulin mutations, reduced function of significant apoptosis proteins (such as Bcl-2 and p53), and alterations in cytokine expression (such as Interleukin-6) [16,17]. The main mechanisms of paclitaxel resistance will be explored and discussed further in this review (Figure 2). 

### 3.1. ABC Transport Mediated MDR in Paclitaxel Chemotherapy

The aforementioned increased drug efflux is mainly due to the action of the molecular ABC transporters present across the cell membrane, employed to “pump out” the substrate specific drug. ABC transporters are naturally occurring, energy dependent transporters which utilize the hydrolysis of ATP to transport respective substrates across the cell membrane. There are many subclasses of these ABC transporters endogenously important in the transport of a variety of substrates. In MDR, the ABC transporters are found to be overexpressed, transporting structurally dissimilar anticancer drugs out of the tumor, reducing the chemotherapeutic efficiency. This review will focus on sub-family B, member 1 (ABCB1) as well as sub-family C, member 10 (ABCC10) and their influence on paclitaxel’s performance in cancer cells. 

Over-expression of ABCB1, also termed MDR1 or P-glycoprotein (P-gp), is one major mechanism of resistance to paclitaxel. The resistance can be a *de novo* or drug-induced mechanism acquired by the cell. ABCB1 is a 170 kDa phosphoglycoprotein encoded by the human MDR1 gene, consisting of two ATP binding cassettes and six transmembrane domains [18]. The transporter is naturally found present in the liver, intestine, kidney, placenta and blood brain barrier [15,19]. The drugs that are primarily transported via ABCB1 are hydrophobic natural products, and include different classes of anticancer drugs such as paclitaxel, doxorubicin, vinblastine and vincristine; as well as more commonly used antiarrhythmic, antihistamine and cholesterol-lowering statin drugs [15]. 

Transporter mediated MDR prompts cross resistance to other drugs which are also substrates of the overexpressed transporter. Cross resistance to paclitaxel has been found in cell lines resistant to alternative anticancer drugs, such as gemcitabine and doxorubicin, due to an increase in MDR mediated efflux [20]. The role of ABCB1 mediated MDR translates to a dire issue in clinical chemotherapy, in the situation of patient chemoresistance leading to refraction of several other drugs. The increased efflux of the chemotherapeutic agents leading to decreased intracellular anticancer drug levels would consequentially lead to ineffective antitumor activity for the patient. 

To overcome the ABCB1 mediated MDR mechanism, investigators have found pre-dosing of small molecule inhibitors, such as kinase inhibitors, with paclitaxel or other ABCB1 substrates to have effective antitumor activity [21,22,23]. First generation found ABCB1/MDR1/P-gp inhibitors, which included the calcium channel blocker verapamil and the immunosuppressant cyclosporine A, showed effective sensitization of cancer cells which were otherwise resistant to paclitaxel, doxorubicin, and other ABCB1 substrates [24,25]. However, these first generation inhibitors materialized in unwarranted toxicities. Second generation P-gp inhibitors, such as valspodar or PSC 833, were later found with increased potency and decreased toxicity [26]. However, the results did not translate to effectiveness in the clinic [27]. Currently, third generation P-gp inhibitors are being investigated, such as tariquidar and zosuquidar. The pharmacodynamics of tariquidar (XR9576) were recently investigated in a clinical trial in combination with docetaxel in 48 patients with lung, ovarian, and cervical cancer. Though the study made clear it was not investigating the clinical benefit of the third generation ABCB1 inhibitor, Kelly and colleagues determined and reported that a docetaxel dose of 75 mg/m^2^ every 3 weeks can be safely administered with a single 150 mg dose of tariquidar; and furthermore, that the study found relatively limited toxicity co-administering this P-gp inhibitor with taxane treatment [28]. The group also reports that two other double-blind, randomized, placebo-controlled, multi-center, phase III trials of combination tariquidar with carboplatin/paclitaxel or vinorelbine to treat advanced NSCLC had been closed prematurely due to toxicity accounts [28]. Through the generations of these inhibitors, we are closer to finding effective treatments to combat ABCB1 mediated chemoresistance. Further toxicity studies of these inhibitors, as well as other compounds, must be done to ensure patient safety. 

Interestingly, other small molecule inhibitors, which were not originally designed to target ABCB1, have been found to circumvent ABCB1 mediated resistance. Sodani and colleges found that the PDE5 inhibitor sildenafil (Viagra^®^) at nontoxic doses, is able to sensitize ABCB1-mediated MDR to paclitaxel *in vitro* and *in vivo*. The group employed human epidermoid carcinoma cell line (KB-3-1) to develop an acquired resistant ABCB1-overexpressing cell line (KB-C2), via increasing exposures to colchicine. Colchicine, as a substrate of ABCB1, is used to induce and maintain the overexpression of the ABCB1 transporter [18]. The pair of cell lines (parental and resistant) were studied *in vitro* and in a mouse xenograft model to show the sensitization effects of sildenafil on ABCB1 mediated resistance to paclitaxel. The PDE5 inhibitor, at non-toxic concentrations at or below 10 uM, demonstrated increased accumulation of paclitaxel, stimulated ATPase activity, and effective sensitization of resistant cells to paclitaxel [23]. Many kinase inhibitors have also been found to reverse ABCB1 mediated resistance [29]. Lapatinib, the dual tyrosine kinase inhibitor which interrupts the epidermal growth factor receptor (EGFR) and human epidermal growth factor receptor 2 (HER2) pathways, has been formulated with paclitaxel in a polyelectrolyte nanocolloid to overcome ABCB2 mediated paclitaxel resistance. Vergara *et al.* have found this combined drug is able to enhance the inhibition of cell growth in the ABCB1 overexpressing ovarian cancer cell line (OVAR-3) compared with paclitaxel alone treatment [30]. This along with other small molecule kinase inhibitors, such as those targeting the vascular endothelial growth factor (VEGF) receptor, should be further investigated *in vivo* for their systemic anti-chemoresistance value and benefit. 

The mechanism by which these inhibitors discussed are able to bind to the ABC transporter and pharmacologically hinder ABCB1 drug efflux is still not fully understood. Recently, McCormick and colleagues have studied the mechanism of ABCB1 *in silico* using targeted molecular dynamics techniques. Employing crystal structures of homologous ABCB1 protein, the docking and transport properties of verapamil, daunorubicin and tariquidar were investigated. Their results show that the transporter substrates docked at different initial binding sites within the cytoplasmic leaflet of the drug binding domains of ABCB1 and were transported through the membrane as the glycoprotein transitioned from an inside-open to an outside-open conformation. The group discovered that tariquidar is able to dock at three locations within ABCB1, either the intracellular loops or at the drug binding domain, and furthermore, that the mechanism by which tariquidar inhibits ABCB1 involves a stabilization of an outward, open conformation [31]. With the tremendous advancements in biotechnology, more *in silico* studies are necessary to further understand *in vitro* and *in vivo* results of ABC transporters on a molecular mechanistic level. This understanding will direct us to discovering safer inhibitors of ABC transporters for therapeutic applications. 

The ABCC transporters comprise a long list of functional lipophilic anion pumps and regulators of ion channels, endogenously located in major organs including the pancreas, colon, spinal cord, lung, trachea, skin, liver, placenta, kidneys, brain, spleen and heart [32,33,34]. ABCC10, also termed human Multidrug Resistance Protein 7 (MRP7), is a 171-kDa protein containing three membrane-spanning domains (MSD1, MSD2 and MSD3) and two NBDs, encoded by the *ABCC10* gene [35]. ABCC10 is known to direct the ATP-dependent transport of 17β-estradiol 17-(β-D-glucuronide) and the glutathione conjugate of leukotriene C4 (LTC4), involved in cellular extrusion of detoxification [36]. The expression of the ABCC10 transporter has been also found to influence anticancer drug resistance [32,37]. A positive correlation of ABCC10 with paclitaxel resistance has been established in NSCLC [38,39]. Gene expression of ABCC10, as well as ABCB1, was found greater in paclitaxel resistant human small cell lung cancer cells (PC-6/TAX1-1) compared to the sensitive, parental cells (PC-6). Furthermore, decreasing the expression of ABCC10 in lung cancer (cell line NCI-H23) by small interfering RNA increased intracellular paclitaxel accumulation, and enhanced paclitaxel cytotoxicity [38]. Although Hopper *et al.* found ABCC10 to have greater influence in docetaxel transport mediated resistance, greater accumulation of paclitaxel was also found in the ABCC10 transfected HEK293 cells [40]. Moreover, *in vitro*, ABCC10 knockout mice were found to be more sensitive to paclitaxel [41]. 

Research using inhibitors of ABCC10 have shown to sensitize resistant cells to paclitaxel. Several small molecule kinase inhibitors have been found to reverse MDR propagated by ABCC10 [39,42,43]. Masitinib, a small molecule stem-cell growth factor receptor (c-Kit) tyrosine kinase inhibitor was found by Kathawala and colleagues to sensitize paclitaxel resistance in HEK293 cells transfected with ABCC10. Inhibiting the ABCC10 transport activity *in vitro*, using non-toxic concentrations of masitinib (2.5 μM), greater intracellular accumulation and decreased efflux of paclitaxel was found. The group also followed with *in vitro* investigation, finding the combination of masitinib with paclitaxel, significantly inhibiting the growth of ABCC10-expressing tumors in nude athymic mice. Pharmacokinetics of the *in vitro* study display the inhibitor administration also resulted in a significant increase in the levels of paclitaxel in the plasma, tumor tissue and lungs of mice, contrary to paclitaxel alone [39]. The role of ABCC10 in transport mediated paclitaxel resistance is important to consider along with ABCB1 in the chemoresistance mechanisms of paclitaxel, and other anticancer drugs. Inhibitors of these ABC transporters may be useful in the clinical setting to overcome transport mediated chemoresistance.

### 3.2. Alterations of the Microtubule Dynamics in Paclitaxel Resistance

Another important paclitaxel resistance mechanism to highlight is the disruption of microtubule dynamics. Normally, tubulin α-β heterodimers are combined in a sequential and polarized manner to form 25 nm hollow tubes. These heterodimer tubes grow out separately in the positive-end direction to the plasma membrane as their minus-ends remain embedded at their organizing center, the centrosome. Cells are able to tolerate minor fluctuations in the tubulin dynamic, but any significant alteration will prevent mitosis and the process cell division [44]. Effective paclitaxel concentrations act by binding to β-tubulin in the inner surface of the microtubule, stabilizing the polymer further decreasing the frequency of detachment, and preventing normal cell division [45]. The alteration of microtubules, whether through tubulin mutations or changes in the expression of microtubule interacting proteins, has been found to limit paclitaxel response both *in vitro* and *in vivo*. 

Paclitaxel insensitivity in cancer cells has been demonstrated by: microtubule mutations (both α and β-tubulin); altered β-tubulin isotype overexpression, specifically isotypes III and V; altered binding of paclitaxel to the microtubule; as well as altered expression or post-translational modifications of microtubule regulatory proteins [17,46,47,48,49]. 

Tubulin (both α and β) mutations seen in paclitaxel resistance can alter the stability of the microtubule network, without affecting the binding affinity of paclitaxel to β-tubulin [45]. Tubulin mutations are found to confer resistance to paclitaxel by increasing the frequency of microtubule detachment and disrupting the spindle function in cell division. A report from 139 isolated Chinese hamster ovary (CHO) cell mutants resistant to paclitaxel found 59 mutants dependent on paclitaxel for normal cell proliferation, and 13 mutants were partially dependent. The two-dimensional gel analysis of whole cell proteins displayed altered tubulin in 13 of the mutants, with six of the 13 harboring altered α-tubulin and seven mutants with altered β-tubulin [50]. A paclitaxel resistant cell line with α-tubulin mutation has also been discovered, which indicates alterations in microtubule function may not be restricted to β-tubulin [51]. Overall, considering the presence of tubulin variations, both α and β, in patient tumors may be of use for the prognosis of paclitaxel response in anticancer therapy. The debate of their influence must be supported by research and clinical studies.

Interestingly, cells selected for resistance to paclitaxel have been found to be cross-resistant to other microtubule stabilizing agents; and furthermore, many of the paclitaxel resistant cells lines have been found to be more sensitive to microtubule destabilizing agents that bind to free tubulin dimers, such as the vinca alkaloids or colchicine [49]. A 2008 study of tumor cells extracted from children treated with vincristine for acute lymphocytic leukemia reported a correlation with vincristine resistance and paclitaxel sensitivity. Although the study neglected to provide genetic information on the presence of a tubulin mutation, xenograft models selected for vincristine resistance were found to have elevated polymer and showed more sensitivity to paclitaxel [52]. The phenotypes reported of increased polymer levels and enhanced paclitaxel sensitivity in vincristine resistant cells, are also congruent with increased vinca alkaloid sensitivity in paclitaxel resistant patient phenotypes for drug resistant cells with mutations in tubulin [45,52]. Monzó *et al.* also published the clinical report identifying β-tubulin gene mutations as a strong predictor of response to paclitaxel in the treatment of NSCLC [53]. Still, further *in vivo* and *ex vivo* investigations are needed to assess the tubulin state in new and recurrent tumors from individual patients before and after chemotherapy to fully ascertain the prevalence and influence of tubulin mutations in the response to paclitaxel and other microtubule targeting agents.

Microtubule interacting proteins have also been found to have altered protein expression in paclitaxel resistant cells. Kinesins are a family of motor protein that travel along microtubules and aid in intracellular transport and mitosis [54]. The kinesin related motor protein MCAK, important in catalyzing the depolymerization of microtubules, has been found to be over-expressed in cancer cells insensitive to paclitaxel [55,56]. The over-expression of these depolymerization motor proteins counteracts paclitaxel’s microtubule polymerization and stabilization function, resisting its anticancer mechanism. Besides the imposition of ABC transporters, microtubule mutations and alterations of their dynamic network, are important concerns and considerations for paclitaxel response in patients. These findings discussed substantiate the role of the variations in the microtubule network as one of the major mechanisms in paclitaxel resistance. Further mechanistic as well as clinical research studies are needed to investigate the impact of variations in microtubule dynamics and tubulin mutations in paclitaxel sensitivity.

## 4. Paclitaxel in Chemoradiotherapy 

The underlying objective of combining chemotherapy and radiotherapy is to improve the therapeutic ratio for patients. Based on the early finding that the peak in radiation sensitivity occurs just before DNA replication begins, it was then theorized and further investigated that paclitaxel may be a potent radiosensitizing agent due to its ability to arrest cells in the G2/M phase of the cell cycle and, therefore, act synergistically when combined with radiation [57,58]. Tishler and colleagues demonstrated this early, examining the effect of drug-radiation exposures on the human astrocytoma (G18) cell line. They reported the sensitizer enhancement ratio (SEM) for 10 nM paclitaxel at 10% survival as approximately 1.8 [58]. Further studies, both *in vitro* and *in vivo*, found improved therapeutic outcome of chemoradiotherapy with paclitaxel [59,60]. A few years later however, in human cervical cancer cells, paclitaxel was said to be a moderate to weak radiation sensitizer [61,62]. Erlich and colleagues revealed that gamma radiation during G2/M phases showed only a small degree of radiosensitization by paclitaxel (10 nM) for the relatively radioresistant MS751 line, but no sensitization using radiation doses of conventional fraction size. The SER values averaged 1.1 and 1.3 for the C-33A and MS751 cell lines, respectively [61]. 

In locally advanced breast cancer (LABC), preoperative chemotherapy is the conventional primary treatment. Formenti *et al.* investigated the efficacy and safety of twice-weekly paclitaxel with concurrent radiation before modified radical mastectomy, followed by adjuvant doxorubicin chemotherapy in 44 patients of stage IIb to III LABC. Results from this phase I/II trial found overall clinical response rate to preoperative paclitaxel and radiation therapy to be 91% from the 44 cases. Toxicities found from paclitaxel with radiation therapy included grade 3 skin desquamation (7%), hypersensitivity (2%), and stomatitis (2%) [63]. Still, the improved overall response rate outweighed the toxicities. 

*In vitro* studies by Liebmann and colleges investigated the radiosensitization properties of paclitaxel in human breast (MCF-7), lung (A549), ovarian (OVG-1), and pancreatic (PC-Sh) adenocarcinoma cells using clonogenic assays and flow cytometry. Though all cell lines were found to arrest at a G2/M phase after exposure to paclitaxel (0 µM to 10 µM), the degree of radiosensitization by paclitaxel varied among the different human cancer cell lines. The SER of paclitaxel at 10% survival was 1.8, 1.6 and 1.5 in the MCF-7, OVG-1 and PC-Sh cells, respectively. The pancreatic cell response of the study showed inconsistencies in the radiosensitization response to paclitaxel. More interestingly, paclitaxel (at any concentration) was unable to enhance the radiation sensitivity of the A549 cells, even when treated with cycloheximide, a protein synthesis inhibitor. The inability of paclitaxel to radiosensitize the human alveolar adenocarcinoma cells, despite the presence cycloheximide, renders that a G2/M arrest is not a necessary condition for paclitaxel radiosensitization [64]. Further research is needed to reveal the Achilles heel of cancer cells for successful chemoradiotherapy, taking into account tissue type and individual patient susceptibility. 

Sunwoo *et al.* presented a human clinical study of concurrent paclitaxel chemotherapy with radiotherapy in 33 previously untreated patients with stage III or IV locally advanced head and neck squamous cell carcinoma (HNSCC) and concluded paclitaxel administered in combination with radiotherapy to be favorable for patients with advanced HNSCC. In the study, paclitaxel was administered as a 120 hour continuous infusion (105 mg/m^2^ and 120 mg/m^2^) every 3 weeks during the course of standard radiation therapy. After three months of therapy, a 76% complete response (CR) at the primary site and a 70% overall CR was achieved. At 36 months, overall survival was 57.8%, and disease-free survival was 51.1%. Though there were local toxicities, including mucositis, dysphagia, and skin reactions, all patients had functional speech, and all but four patients were swallowing food within 3 months post treatment. Interestingly, the steady-state plasma concentrations of paclitaxel were not reached during the 120 hour infusion [65]. This finding suggests the chemoradiotherapy to be a non-linear process and prompts further cell cycle investigation. 

A recent 2015 meta-analysis performed by Zhang and colleagues investigated cervical cancer treatments and the efficacy of different concurrent chemoradiotherapy. Zhang and colleges determined paclitaxel as one of the chemotherapies to have a better effect on reducing chemotherapy toxicity, compared to cisplatin. The meta-analysis indicated that the future of concurrent chemoradiotherapy may be directed towards radiation concurrent with non-cisplatin but another single drug, as to alleviate adverse side effects for the patient [66]. 

With combined chemoradiotherapy treatment regimens, it is vital to seek the greatest therapeutic index for patients. Although it is widely used, paclitaxel is found to have several adverse effects such as peripheral sensory neuropathy and cutaneous toxicity, especially when concurrent with radiation therapy [67,68]. To enhance the pharmacokinetics of paclitaxel while limiting the toxicities, novel polymeric micelle nanocarrier formulations have been prepared for tumor-targeted delivery of paclitaxel [69,70]. The intriguing nature of these micelle nanocarriers is that they can be altered to encapsulate large amounts of the hydrophobic drug while maintaining continued circulation [71]. Negishi and colleagues published data stating NK105, a paclitaxel-incorporating micellar nanoparticle, is a more potent radiosensitising agent as compared to free paclitaxel [72]. Hamaguchi and colleagues found that with NK105, a paclitaxel-entrapped micellar nanoparticle (85 nm), the deposition of paclitaxel in tumors was increased, while the drug exposure to normal tissue was reduced [69]. The study used Lewis lung carcinoma (LLC) tumor xenograft mice to identify the antitumor activity of NK105 *versus* paclitaxel alone, incorporating concurrent radiation therapy as well. After 24 hours, the NK105-treated tumor cells showed a stronger trend of arrest at the G2/M phase than the paclitaxel alone treated cells. On day 9 of the concurrent radiation treatment study, the combined NK105 therapy with radiation yielded greater antitumor activity as compared to both the radiotherapy and combined paclitaxel chemoradiotherapy groups [69]. Notably, the mice across the groups showed no significant differences in weight, and therefore no visible significant differences in toxicities. These nanocarrier formulations and novel pharmacokinetics are exciting and promising as a new administration of chemotherapy for the betterment of cancer patients. Combined with radiation, a synergistic anticancer effect could be achieved in the patient.

## 5. Conclusions 

Paclitaxel has been a prominent player in anticancer treatment. As an effective microtubule stabilizer and radiation sensitizer, paclitaxel has been applied to many anticancer treatment regimens. However, the therapeutic efficacy of paclitaxel has been found to induce multidrug resistance (MDR) through various cellular mechanisms which are still not fully understood. Besides overexpression of the ABCB1 and ABCC10 efflux transporters, paclitaxel resistance has also found to include both α and β tubulin mutations, alterations in the binding regions of β-tubulin, reduced function of important proteins involved in apoptosis, as well as alterations in cytokine expression. Novel drug delivery systems, including nanoparticles and targeted drug conjugates, will allow paclitaxel to find its way to the tumor tissue for more direct and safe anticancer activity. Furthermore, new compounds and even currently approved small molecule inhibitors must be explored to overcome the ABC transport mediated MDR accompanying paclitaxel resistance. It has been over 40 years since the isolation and identification of paclitaxel (Taxol^®^), and its unique pharmacokinetic and pharmacodynamics anticancer properties still uphold its significance in the clinical treatment of cancer. With more research aimed at uncovering the origination of *de novo* and acquired resistance, as well as more effective chemoradiotherapy, paclitaxel treatment will continue to transcend the ages of anticancer therapy. 
